# Altered Regional Brain Activity and Functional Connectivity Between Non‐Diabetic and Diabetic Kidney Disease: A Resting‐State fMRI Study

**DOI:** 10.1002/brb3.71368

**Published:** 2026-04-15

**Authors:** Xiwen Lei, Yiyan Sun, Juan Li, Shuang He, Yangjie Yu, Xuyun Hua, Junjie Pan, Rui Dong, Jianguang Xu

**Affiliations:** ^1^ Department of Pathology The PLA Naval Medical University Shanghai Changhai Hospital Shanghai China; ^2^ School of Rehabilitation Science Shanghai University of Traditional Chinese Medicine Shanghai China; ^3^ Department of Nephrology The PLA Naval Medical University Shanghai Changhai Hospital Shanghai China; ^4^ Shuguang Hospital Affiliated to Shanghai University of Traditional Chinese Medicine Shanghai China; ^5^ Department of Cardiology Huashan Hospital Fudan University Shanghai China; ^6^ Engineering Research Center of Traditional Chinese Medicine Intelligent Rehabilitation Ministry of Education Shanghai China; ^7^ Department of Orthopedics Yueyang Hospital of Integrated Traditional Chinese and Western Medicine Shanghai University of Traditional Chinese Medicine Shanghai China; ^8^ Department of Rehabilitation Medicine Yueyang Hospital of Integrated Traditional Chinese and Western Medicine Shanghai University of Traditional Chinese Medicine Shanghai China

**Keywords:** amplitude of low‐frequency fluctuations, diabetic kidney disease, functional connectivity, resting‐state functional magnetic resonance imaging

## Abstract

**Introduction:**

Patients with chronic kidney disease often exhibit impaired brain function; however, the differences between those with non‐diabetic kidney disease (non‐DKD) and diabetic kidney disease (DKD) remain poorly understood. This study aimed to investigate alterations in resting‐state brain activity in patients with non‐DKD and DKD.

**Methods:**

Thirty non‐DKD patients, thirty DKD patients, and twenty‐nine healthy controls underwent laboratory examinations and resting‐state functional magnetic resonance imaging (rs‐fMRI). The brain activity was analyzed using the amplitude of low‐frequency fluctuations (ALFF) and seed‐based functional connectivity (FC), and correlations between laboratory indicators and FC were examined.

**Results:**

Both non‐DKD and DKD groups exhibited reduced ALFF in several brain regions, including the bilateral putamen, alongside elevated ALFF in the left middle occipital gyrus. Seed‐based FC analysis revealed decreased connectivity between bilateral putamen and several regions, including decreased FC between the left putamen and left caudate. Compared with the non‐DKD group, the DKD group demonstrated reduced ALFF in the left putamen and right precuneus, along with decreased FC between the right putamen and right thalamus. Several biomarkers, including urinary protein‐to‐creatinine ratio (UPCR), C‐reactive protein (CRP), and hemoglobin (HGB), were associated with observed FC alterations.

**Conclusion:**

Our findings indicate that DKD patients exhibit distinct patterns of brain activity compared to non‐DKD patients, with the putamen potentially acting as a key neural target in the progression of CKD. These functional alterations correlate closely with systemic status, suggesting a significant role in the pathogenesis of neural impairment. Our results may enhance the understanding of neural functional alterations and the underlying mechanisms in non‐DKD and DKD patients.

## Graphical abstract

This resting‐state functional MRI (rs‐fMRI) study identifies distinct patterns of spontaneous brain activity in patients with diabetic kidney disease (DKD) compared to non‐diabetic kidney disease (non‐DKD). Altered functional connectivity (FC) between the putamen and specific brain regions implicates its potential significance as a key neural target in chronic kidney disease (CKD). Furthermore, hemoglobin levels exhibit a significant association with neural dysfunction in patients with DKD.

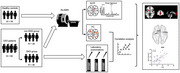

## 1 Introduction

Chronic kidney disease (CKD) is a major public health issue, with a global prevalence of 8.2% in China (Wang et al. 2023). CKD resulting from diabetes accounts for one‐third of all disability‐adjusted life years (DALYs) (Kidney Disease: Improving Global Outcomes (KDIGO) CKD Work Group 2024). Previous research has demonstrated an association between CKD and altered brain function, including impaired global topological network properties in affected patients (Song et al. 2023). Diabetic kidney disease (DKD), a major cause of CKD, is associated with a high prevalence of cardiovascular comorbidities and imposes a substantial economic burden (Gupta et al. 2023; Folkerts et al. 2020). While uremia‐related brain injury is a shared feature of both conditions, hyperglycemia in DKD uniquely induces proinflammatory astrocyte reprogramming and blood‐brain barrier dysfunction, potentially leading to functional brain patterns distinct from those observed in non‐DKD cases (Lee et al. 2024). Therefore, elucidating the specific neuropathological mechanisms in DKD is crucial for guiding early interventions and improving clinical outcomes.

Resting‐state functional magnetic resonance imaging (rs‐fMRI) provides a noninvasive and promising approach for elucidating the neurobiological underpinnings of cerebral functional changes. The amplitude of low‐frequency fluctuations (ALFF) reflects regional brain activity by assessing the power of the blood oxygen level‐dependent (BOLD) signal within a specific low‐frequency range (0.01–0.08 Hz) (Zou et al. 2008); however, it does not indicate functional connectivity (FC) between brain regions. To explore these connections, seed‐based FC analysis can be employed, providing an intuitive interpretation of the results (Lv et al. 2018). Numerous neuroimaging studies have confirmed that alterations in brain functional networks are common across a range of diseases (Benedict et al. 2020; Chen et al. 2024; Ibrahim et al. 2021). Yin et al. reported that patients with end‐stage kidney disease (ESKD) exhibit deficits in decision‐making processes as well as impairments in the associated neural network (2025). Our previous study has also demonstrated altered ALFF and regional homogeneity (ReHo) values in specific brain regions of CKD patients (Yu et al. 2024). Moreover, type 2 diabetes mellitus (DM) has been shown to independently alter cerebral glucose metabolism and global brain network assortativity (Li et al. 2022). Given that diabetes exerts a distinct influence on neural activity, it is hypothesized that patients with CKD and concurrent diabetes (DKD) exhibit different patterns of brain activity compared to those without diabetes (non‐DKD). However, to our knowledge, no studies have explored the differences in spontaneous brain activity changes between non‐DKD and DKD patients. Understanding these underlying mechanisms may help ameliorate the neurological conditions of patients with CKD and facilitate the design of early and effective clinical interventions to prevent disease progression.

Therefore, this study aims to investigate and compare the alterations of brain activity using ALFF and FC analysis between non‐DKD and DKD patients.

## 2 Materials and Methods

### 2.1 Participants

This controlled study was approved by the ethics committee of Shanghai Changhai Hospital, Naval Medical University (No. CHEC2022‐159). All study procedures follow the ethical principles for medical research involving human subjects of the Declaration of Helsinki. A total of 30 non‐DKD patients (15 males and 15 females; aged 58.53 ± 14.15 years), 30 DKD patients (18 males and 12 females; aged 55.73 ± 12.21 years), and 29 healthy controls (HCs; 13 males and 16 females; aged 58.14 ± 13.44 years) were enrolled from August 1, 2020, to May 31, 2023.

The inclusion criteria are as follows: (1) right‐handed; (2) aged 30–79 years; (3) for CKD participants, a glomerular filtration rate of less than 60 mL/min/1.72 m^2^; (4) For DKD participants, CKD with albuminuria (urinary albumin‐to‐creatinine ratio ≥30 mg/g in ≥2 tests over 3–6 months) and a history of diabetes or renal biopsy‐confirmed diabetic nephropathy (DN) pathology; (5) CKD duration >1 year; (6) the ability to complete the MRI examination; (7) the provision of written informed consent. The exclusion criteria are as follows: (1) active glomerulonephritis or immunosuppressive therapy; (2) receiving any renal replacement therapy (hemodialysis, peritoneal dialysis, or kidney transplantation); (3) malignancy, infectious diseases, or pyrexia; (4) any brain lesions such as strokes; (5) any neuropsychiatric diseases that impair neurological function, such as depression and Alzheimer's disease; (6) major cardiovascular, respiratory, or gastrointestinal disorders; (7) MRI contraindications; (8) for non‐DKD participants, a history of diabetes.

### 2.2 Clinical Evaluations and Laboratory Examination

All participants underwent blood biochemistry tests before MRI scanning. A comprehensive set of laboratory indicators was collected, including hemoglobin A1c (HbA1c, %), blood urea nitrogen (BUN, mmol/L), serum creatinine (SCr, µmol/L), estimated glomerular filtration rate (eGFR, mL/min/1.73 m^2^), carbon dioxide combining power (CO_2_CP, mmol/L), urinary albumin‐to‐creatinine ratio (UACR, mg/g), urinary protein‐to‐creatinine ratio (UPCR, mg/g), serum calcium (mmol/L), serum phosphorus (P, mmol/L), white blood cell count (WBC, ×10^9^/L), lymphocyte percentage (Lym, %), hemoglobin (HGB, g/dL), serum albumin (ALB, g/dL), C‐reactive protein (CRP, mg/L), erythrocyte sedimentation rate (ESR, mm/h), parathyroid hormone (PTH, pg/mL), 24‐h urinary protein (UP, mg/24 h), and B‐type natriuretic peptide (BNP, pg/mL).

### 2.3 Rs‐fMRI Scanning

A 3T Magnetom Trio A MR Scanner (Siemens AG, Erlangen, Germany) at Changhai Hospital was used for image acquisition. All participants were instructed to remain awake in a resting state with eyes closed, refraining from active thinking. Head movement was restricted using foam pads. Rs‐fMRI was performed using a gradient‐echo echo‐planar imaging (GRE‐EPI) sequence with the following parameters: field of view = 240 × 240 mm^2^, matrix size = 64 × 64, slice number = 31, spacing between slices = 4.5, and the scanning order was interleaved; slice thickness = 3.6 mm, acquisition voxel size = 3.5 × 3.5 × 4.5 mm^3^; flip angle = 90°, repetition time/echo time = 2000/30 ms, and there were 240 volumes.

### 2.4 Data Processing and Calculation of Amplitude of Low‐Frequency Fluctuation

Resting‐state fMRI data were processed with the Resting‐State fMRI Data Analysis Toolkit plus V1.31 (RESTplus V1.31) (http://restfmri.net/forum/restplus) based on MATLAB (https://ww2.mathworks.cn/products/matlab.html) and Statistical Parametric Mapping 12 (SPM12) (http://fil.ion.ucl.ac.uk.spm/), followed by ALFF and seed‐based FC analyses. Main processing steps include (1) manual reorientation of the structural and functional images so that the anterior commissure lies on the origin ([0, 0, 0]‐coordinate) (Borgan et al. 2019); (2) removal of the initial 10 volumes; (3) slice‐timing; (4) head movement correction (exclusion threshold: > 2° or 2 mm); (5) spatial normalization to Montreal Neurological Institute (MNI) standard template space; (6) 6‐mm Gaussian kernel smoothing; (7) temporal detrending; and (8) regressing out of covariates, including white matter, cerebrospinal fluid, and the first 24 head movement parameters. For the ALFF analysis, bandpass filtering (0.01–0.08 Hz) was applied, followed by Fisher's z‐transformation of the resulting data for subsequent analyses.

### 2.5 Seed‐based Functional Connectivity Analysis

Based on statistically significant clusters identified through z‐scored ALFF (zALFF) group comparisons and supported by prior evidence (Yu et al. 2024), we hypothesized that the bilateral putamen plays a critical role in CKD. Accordingly, we extracted the putamen regions from the significant clusters (Cluster 1 and Cluster 2) centered on the bilateral putamen to serve as seed points for seed‐based FC analysis. The resultant brain maps were then Fisher z‐transformed for subsequent statistical analyses.

### 2.6 Statistical Analysis

#### 2.6.1 Statistical Analysis of Clinical and Laboratory Data

Statistical analysis of clinical and laboratory parameters was performed with SPSS Statistics (version 20.0). The normality of data distribution was assessed using the Shapiro–Wilk test. Age and body mass index (BMI) were expressed as mean ± standard deviation and compared with a one‐way analysis of variance (ANOVA). Sex was compared using the chi‐square test. Education level differences across the three groups were assessed with the Kruskal‐Wallis test. Normally distributed laboratory indicators were analyzed using *t*‐tests, whereas Mann–Whitney *U* tests were applied to non‐normally distributed variables to compare the non‐DKD and DKD groups.

#### 2.6.2 Statistical Analysis of Neuroimaging Data

Differences in ALFF and FC among the DKD, non‐DKD, and HC groups were evaluated using an analysis of covariance (ANCOVA) in SPM12 within the MATLAB environment, with age, sex, and BMI included as covariates. Statistical significance was established at a voxel‐level threshold of *p* < 0.001 (uncorrected) and a cluster‐level threshold of *p* < 0.05, corrected for multiple comparisons using the Benjamini–Hochberg false discovery rate (FDR). For brain regions exhibiting significant group differences, the ALFF and FC values were extracted for post hoc pairwise comparisons (FDR‐corrected). To ensure the robustness of these findings, a permutation test with 5000 iterations was performed. During each iteration, group labels were randomly shuffled—preserving the original covariate structure—and the ANCOVA and FDR procedures were repeated to record the number of significant clusters. The observed results were then compared against this null distribution to determine the probability of the findings occurring by chance.

#### 2.6.3 Correlation Analysis

To assess the clinical significance of altered FC, we conducted correlation analyses between FC indices and clinical parameters. Pearson or Spearman correlation analyses were employed depending on the normality of the data distribution. Multiple comparison corrections were implemented using the FDR method, applied independently to each clinical parameter. The robustness of these associations was validated via a permutation test (5,000 iterations). For each iteration, clinical parameters were randomly shuffled, and the entire correlation and FDR correction pipeline was repeated. A permutation‐based *p*‐value was subsequently derived by comparing the observed number of significant correlations against the resulting null distribution.

## 3 Results

### 3.1 Demographic and Clinical Characteristics

We included data from 89 participants in our study, including 30 patients in the non‐DKD group, 30 patients in the DKD group, and 29 patients in the HC group. The baseline information of the three groups is summarized in Table 1. Demographic characteristics (sex, age, and education status) showed no significant intergroup differences (all *p* > 0.05), except for higher BMI in the DKD group compared with both the non‐DKD and HC groups (*p* = 0.025).

**TABLE 1:** Demographic and clinical characteristics of the study participants.

Variable	HC	CKD		Statistical value	*p*‐value
		Non‐DKD	DKD		
**Demographic characteristics**					
Age, y	58.14 ± 13.44	58.53 ± 14.15	55.73 ± 12.21	*F* = 0.389	0.679
Men, *n* (%)	13 (44.83)	15 (50.00)	18 (60.00)	χ^2^ = 1.411	0.494
BMI, kg/m^2^	24.23 ± 1.85	23.86 ± 4.28	26.21±3.88	*F* = 3.847	0.025^*^
Education, y	6.00(6.00, 9.00)	9.00 (6.00, 12.00)	9.00 (6.00, 12.75)	*H* = 4.834	0.089
**Laboratory indicators**					
HbA1c, %	N/A	5.50 (5.15, 6.00)	6.75 (5.88,7.78)	*Z* = −4.349	< 0.001
BUN, mmol/L	N/A	16.50 (13.98, 25.28)	17.30 (11.50, 22.93)	*Z* = −0.384	0.701
SCr, µmol/L	N/A	352.50 (219.75, 588.50)	300.50 (198.00, 532.50)	*Z* = −0.643	0.520
eGFR, mL/min/1.73m^2^	N/A	13.00 (7.88, 23.05)	15.65 (10.08, 27.45)	*Z* = −0.924	0.355
CO_2_CP, mmol/L	N/A	20.30±3.09	21.27±3.02	*t* = −1.227	0.225
UACR, mg/g	N/A	1108.00(543.75, 1436.50)	3922.05(2729.75,5241.25)	*Z* = −5.589	< 0.001
UPCR, mg/g	N/A	1755.48±1199.21	5948.86±3383.67	*t* = −6.398	< 0.001
Ca^2+^, mmol/L	N/A	2.19(2.12, 2.29)	2.04(1.90,2.18)	*Z* = −2.951	0.003
P, mmol/L	N/A	1.44(1.33,1.68)	1.44(1.27,1.74)	*Z* = −0.118	0.906
WBC, ×10^9^/L	N/A	6.05(4.14,7.20)	6.25(5.20,7.54)	*Z* = −1.360	0.174
Lym, %	N/A	24.97 ± 7.75	21.77 ± 6.344	*t* = 1.749	0.086
HGB, g/dL	N/A	103.70 ± 23.94	105.23 ± 24.82	t = −0.244	0.808
ALB, g/dL	N/A	39.07 ± 5.00	32.70 ± 5.99	*t* = 4.468	< 0.001
CRP, mg/L	N/A	2.54 (1.50, 3.88)	3.02 (1.58, 6.11)	*Z* = −1.039	0.299
ESR, mm/h	N/A	30.50 (22.00, 44.75)	42.00 (24.00, 63.75)	*Z* = −1.665	0.096
PTH, pg/mL	N/A	104.50 (46.53, 240.25)	149.00 (80.70, 199.75)	*Z* = −0.731	0.465
Up, mg/24h	N/A	1621.20 (873.15, 2796.25)	4539.20 (3196.45, 6174.50)	*Z* = −4.844	< 0.001
BNP, pg/mL	N/A	49.32 (24.42, 105.60)	133.74 (24.25, 412.29)	*Z* = −1.700	0.089

*Post hoc analysis revealed statistically significant differences between the DKD group and both the non‐DKD group and the HC group.

Abbreviations: *x* ^2^, chi‐squared test; ALB, serum albumin; BMI, Body Mass Index; BNP, B‐type natriuretic peptide; BUN, blood urea nitrogen; Ca^2+^, serum calcium; CKD, chronic kidney disease; CO_2_CP, carbon dioxide combining power; CRP, C‐reactive protein; DKD, diabetic kidney disease; eGFR, estimated glomerular filtration rate; ESR, erythrocyte sedimentation rate; F, one‐way analysis of variance; H, Kruskal–Wallis *H* test; HbA1c, hemoglobin A1c; HC, healthy control; HGB, hemoglobin; Lym, lymphocyte Percentage; N/A, not applicable; non‐DKD, non‐diabetic kidney disease; P, serum phosphorus; PTH, parathyroid hormone; SCr, serum creatinine; t, two‐sample *t*‐test; UACR, urinary albumin‐to‐creatinine ratio; Up, 24‐h urinary protein; UPCR, urinary protein‐to‐creatinine ratio; WBC, white blood cell count; Z, Mann–Whitney *U* test.

Compared with the non‐DKD group, patients with DKD had significantly higher HbA1c (6.75 [5.88‐7.78] versus 5.50 [5.15‐6.00], *p* < 0.001), higher UACR (3922.05 [2729.75‐5241.25] versus 1108.00 [543.75‐1436.50], *p* < 0.001), higher UPCR (5948.86 ± 3383.67 versus 1755.48 ± 1199.21, *p* < 0.001), higher UP (4539.20 [3196.45‐6174.50] versus 1621.20 [873.15‐2796.25], *p* < 0.001), lower serum calcium (2.04 [1.90‐2.18] versus 2.19 [2.12‐2.29], *p* = 0.003) and lower ALB (32.70 ± 5.99 versus 39.07 ± 5.00, *p* < 0.001) (Table 1).

### 3.2 Comparison of Amplitude of Low‐Frequency Fluctuations Between Non‐DKD, DKD and HC Groups

**FIGURE 1:**
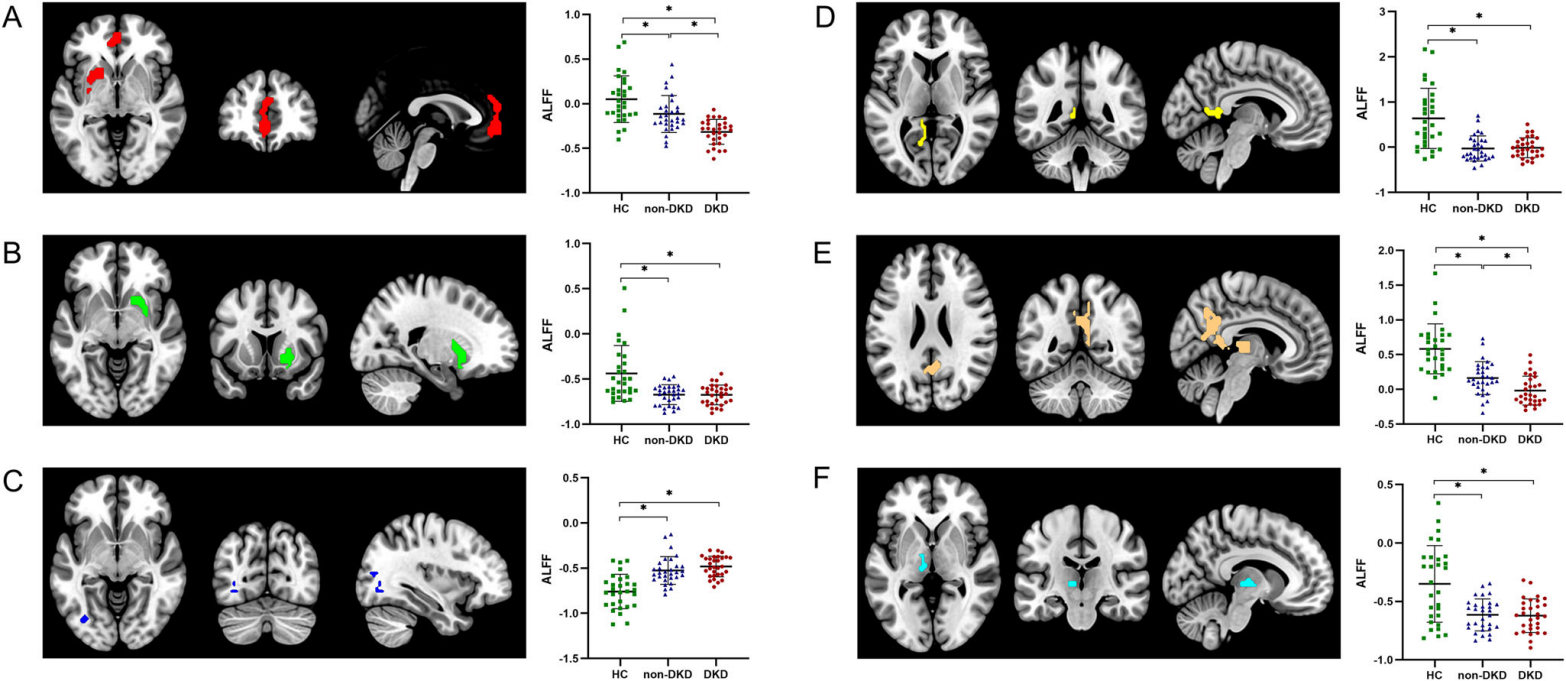
ALFF differences among the HC, non‐DKD, and DKD groups (FDR corrected) and pairwise comparison results. (A) The brain region distribution of significant cluster 1 with Putamen_L as the main brain region and pairwise comparisons of ALFF values within the cluster, (B) The brain region distribution of significant cluster 2 with Putamen_R as the main brain region and pairwise comparisons of ALFF values within the cluster, (C) The brain region distribution of significant cluster 3 with Occipital_Mid_L as the main brain region and pairwise comparisons of ALFF values within the cluster, (D) The brain region distribution of significant cluster 4 with Calcarine_L as the main brain region and pairwise comparisons of ALFF values within the cluster, (E) The brain region distribution of significant cluster 5 with Precuneus_R as the main brain region and pairwise comparisons of ALFF values within the cluster, and (F) The brain region distribution of significant cluster 1 with Thalamus_L as the main brain region and pairwise comparisons of ALFF values within the cluster. *Pairwise comparisons show statistical differences after False Discovery Rate (FDR) correction.

Compared with the HC group, both the non‐DKD and DKD groups exhibited significantly lower ALFF values in the bilateral putamen (non‐DKD: left *p* = 0.003, right *p* < 0.001; DKD: left *p* < 0.001, right *p* < 0.001), left calcarine cortex (*p* < 0.001), right precuneus (*p* < 0.001), and left thalamus (*p* < 0.001), but higher ALFF values in the left middle occipital gyrus (*p* < 0.001) (Table 2, Figure 1A‐F). Compared to the non‐DKD group, the DKD group showed significantly reduced ALFF values in the left putamen (*p* < 0.001) and right precuneus (*p* = 0.004) (Figure 1A, E). All findings remained significant following FDR correction. Permutation testing confirmed the robustness of these ALFF findings (permutation *p* = 0.001).

**TABLE 2:** ALFF alterations among HC, non‐DKD, and DKD group.

Cluster	Structure name	Voxel size	*F*‐value	MNI peak coordinates		
				x	y	z
1	Putamen_L, Cingulum_Ant_L, Frontal_Med_Orb_L	345	19.561	0	45	−3
2	Putamen_R	106	16.646	21	12	−6
3	Occipital_Mid_L	40	13.695	−33	−72	−6
4	Calcarine_L, Precuneus_L	49	21.503	−6	−45	6
5	Precuneus_R, Thalamus_R	289	21.692	6	−51	24
6	Thalamus_L	34	13.125	−9	−21	0

Abbreviations: ALFF, amplitude of low frequency fluctuations; DKD, diabetic kidney disease; HC, health control; L, left; non‐DKD, Non‐diabetic kidney disease; R, right.

### 3.3 Seed‐based FC Analysis

**FIGURE 2:**
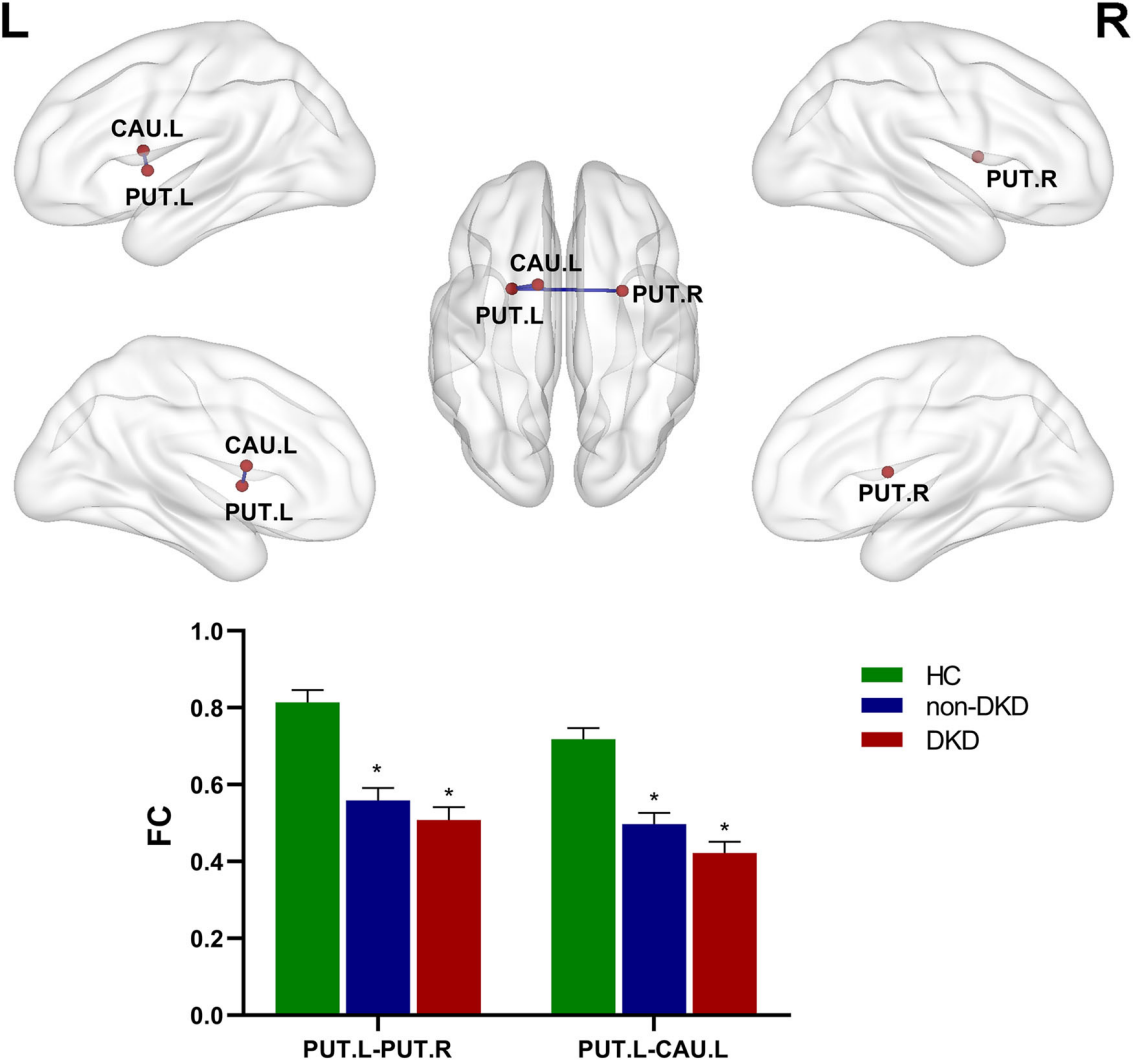
Results of FC analysis selecting PUT.L as the seed point. *Compared with the HC group, *p* < 0.05 (FDR‐corrected).

**FIGURE 3:**
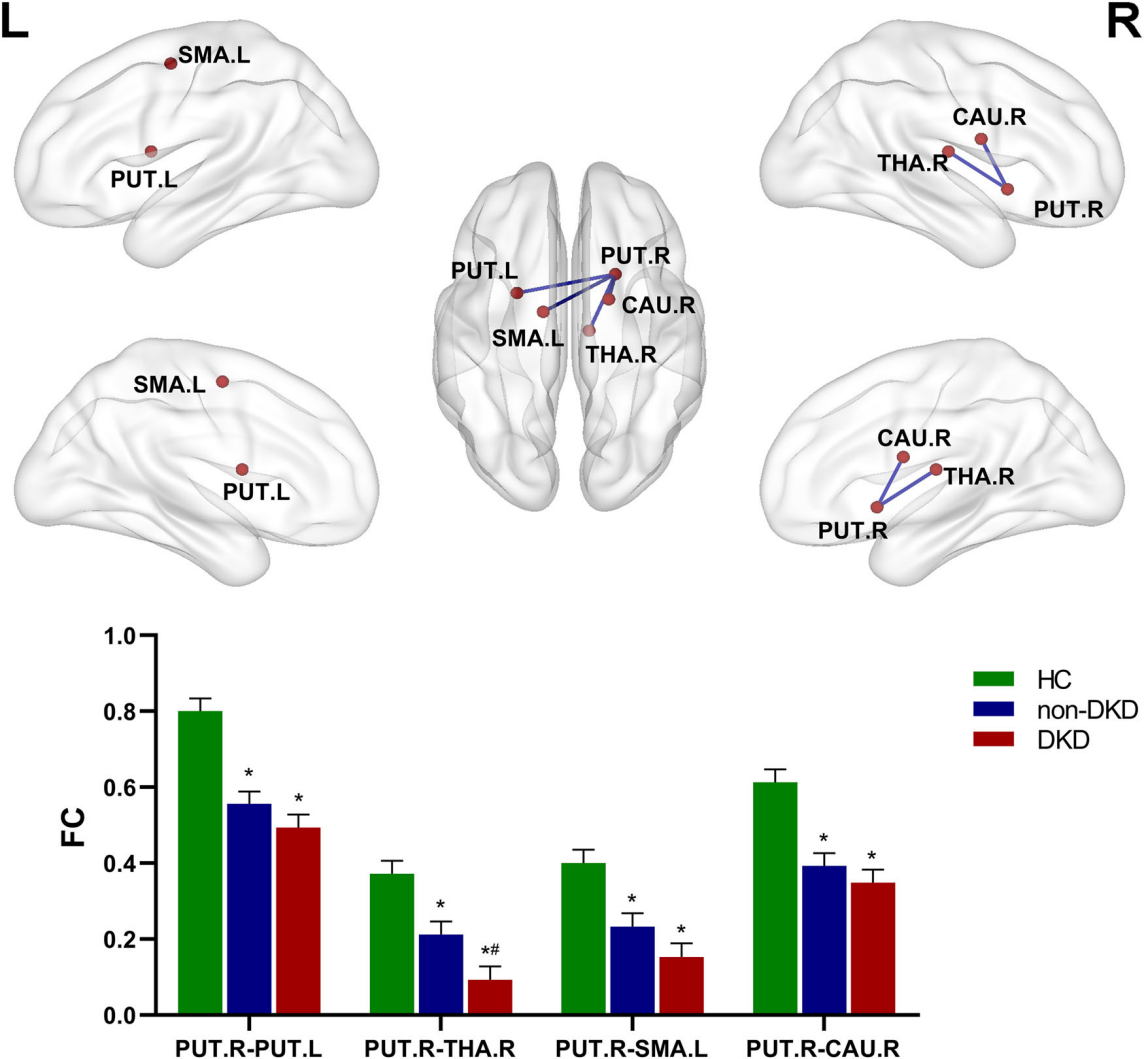
Results of FC analysis selecting PUT.R as the seed point. *Compared with the HC group, *P* < 0.05 (FDR‐corrected). #Compared with the non‐DKD group, *P* < 0.05 (FDR‐corrected).

Significant differences in FC between the bilateral putamen and several brain regions were observed across the three groups. Post hoc analyses revealed that for the left putamen, FC values in the right putamen and left caudate were significantly lower in both the non‐DKD and DKD groups compared to the HC group (*p* < 0.001) (Table 3, Figure 2). Similarly, for the right putamen, the FC values in the left putamen, right thalamus, left supplementary motor area (SMA), and right caudate were significantly reduced in both the non‐DKD and DKD groups relative to controls (*p* < 0.01) (Table 3, Figure 3). Furthermore, the DKD group showed significantly decreased FC between the right putamen and right thalamus compared to the non‐DKD group (*p* = 0.020) (Figure 3). All findings remained significant following FDR correction. Permutation testing confirmed the robustness of these FC findings (permutation *p* = 0.0016).

**TABLE 3:** FC alterations among HC, non‐DKD, and DKD group.

Connected region	Peak areas	Voxel size	*F*‐value	MNI peak coordinates		
				x	y	z
Seed1‐Putamen_L	Putamen_R	358	24.581	27	3	9
	Caudate_L	100	22.670	−12	6	12
Seed2‐Putamen_R	Putamen_L	392	25.018	−24	3	12
	Thalamus_R	80	15.585	9	−15	12
	Supp_Motor_Area_L	78	18.709	−12	−6	54
	Caudate_R	67	15.360	18	0	18

Abbreviations: DKD, diabetic kidney disease;FC, functional connectivity; HC, health control; L, left; non‐DKD, non‐diabetic kidney disease; R, right.

### 3.4 Associations Between FC and Clinical Characteristics

**FIGURE 4:**
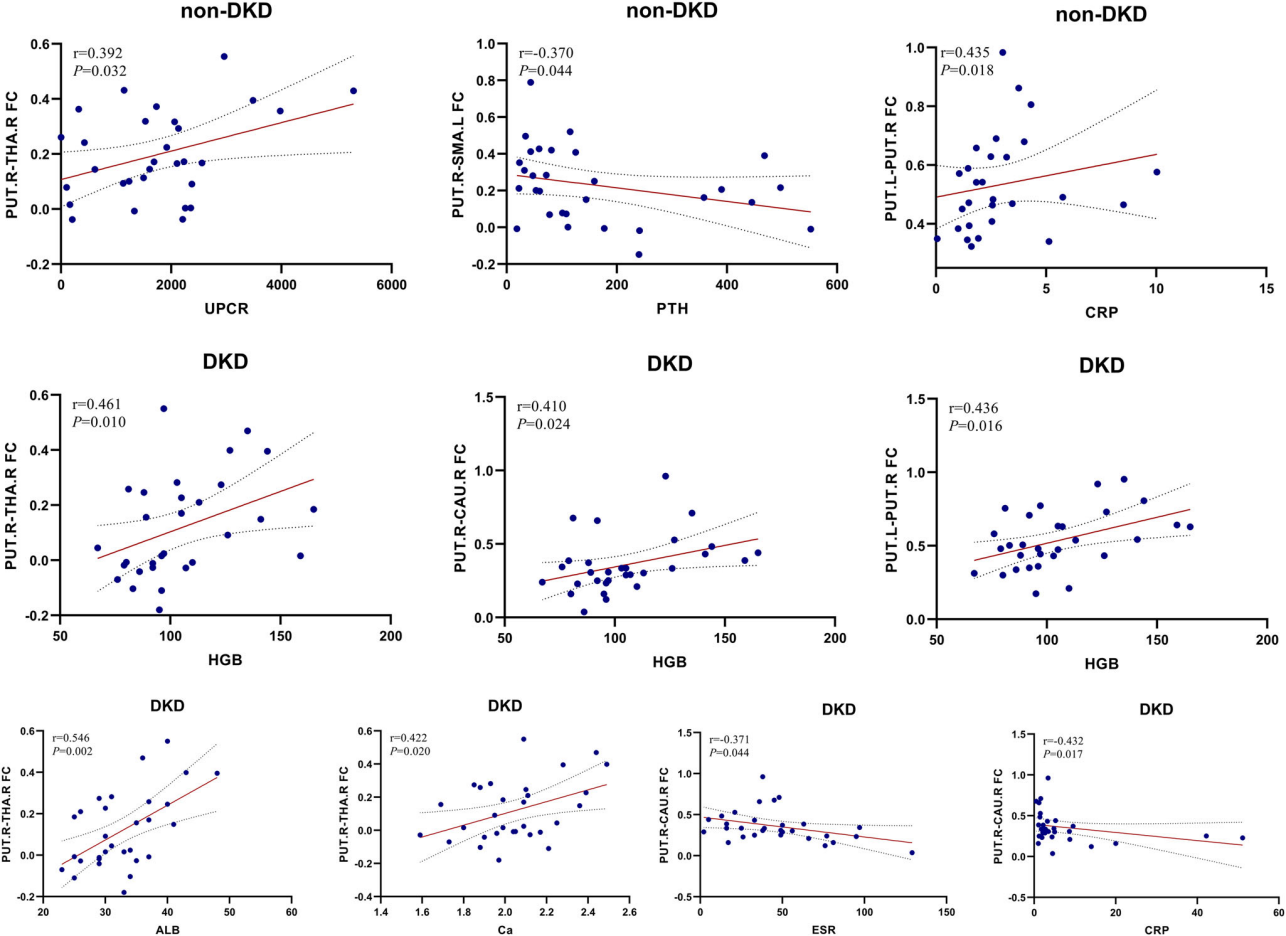
Correlation of FC with laboratory indicators in non‐DKD and DKD patients. Asterisks indicate significant correlations after False Discovery Rate (FDR) correction (*p < 0.05). Abbreviations: ALB, serum albumin; Ca, serum calcium; CRP, C‐reactive protein; DKD, diabetic kidney disease; ESR, erythrocyte sedimentation rate; FC, functional connectivity; HGB, hemoglobin; non‐DKD, non‐diabetic kidney disease; PTH, parathyroid hormone; UPCR, urinary protein‐to‐creatinine ratio.

In the non‐DKD group, significant positive correlations were observed between UPCR and FC values of the right putamen and right thalamus (*r* = 0.392, *p* = 0.032), as well as between CRP and FC values of the left putamen and right putamen (*r* = 0.435, *p* = 0.018), whereas a negative correlation was found between PTH and FC values of the right putamen and left SMA (*r* = −0.370, *p* = 0.044) (Figure 4).

In the DKD group, HGB showed significant positive correlations with FC values of the right putamen and right thalamus (*r* = 0.461, *p* = 0.010), right putamen and right caudate (*r* = 0.410, *p* = 0.024), and left putamen and right putamen (*r* = 0.436, *p* = 0.016). Additionally, ALB positively correlated with FC values of the right putamen and right thalamus (*r* = 0.546, *p* = 0.002), while serum calcium exhibited a similar positive association (*r* = 0.422, *p* = 0.020). Conversely, ESR and CRP demonstrated significant negative correlations with FC values of the right putamen and right caudate (ESR: r = −0.371, *p* = 0.044; CRP: *r* = −0.432, *p* = 0.017) (Figure 4). Correlations surviving FDR correction are marked with asterisks in Figure 4.

Permutation testing (5000 iterations) indicated that the number of significant correlations identified in the primary analysis had a 15.9% probability of occurring by chance (permutation *p* = 0.159). Consequently, the observed global pattern of correlations did not significantly exceed expectations under the null hypothesis. Therefore, these correlations should be interpreted as exploratory in nature.

## 4 Discussion

In this study, we used ALFF and FC to explore the spatial patterns in non‐DKD and DKD patients. A total of 30 non‐DKD patients, 30 DKD patients, and 29 age‐ and sex‐matched HCs were ultimately included. Abnormalities in the ALFF values were identified in several brain regions, including reduced ALFF values in the bilateral putamen, which is consistent with our previous study (Yu et al. 2024). Functional connectivity changes were observed between the bilateral putamen and specific brain regions. Additionally, we found several clinical laboratory indices showing significant correlations with the FC between the putamen and some brain regions in both the non‐DKD and DKD groups.

ALFF is a neuroimaging measure used in resting‐state fMRI to assess spontaneous brain activity (Zou et al. 2008). Compared to HCs, both the non‐DKD and DKD groups exhibited decreased ALFF values in several brain regions, including the bilateral putamen, right precuneus, left thalamus, and left calcarine cortex, along with increased ALFF values in the left middle occipital gyrus. This aligns with our previous findings and those of Song et al. (2024) who reported increased ALFF values in the left middle occipital gyrus and decreased values in the right precuneus in CKD stages 3b‐5 compared to HCs. These findings could enrich our understanding of the intricate relationship within the kidney‐brain axis that drives cognitive and motor impairments in CKD. Notably, the left middle occipital gyrus showed increased ALFF values in both the non‐DKD and DKD groups. This region, located in the lateral occipital lobe, is part of the visual cortex (Destrieux et al. 2010). Given that patients with CKD face an elevated risk of visual impairment due to hypertension or ocular conditions associated with uremia (Nusinovici et al. 2019; Zhu et al. 2020), we hypothesize that the increased ALFF values in this region may reflect a compensatory mechanism to maintain normal visual function. Of particular interest, the ALFF values in the left putamen and right precuneus were significantly lower in the DKD group compared to the non‐DKD group, indicating that DM exerts an influence on these brain regions in patients with CKD. The putamen is a critical region associated with brain atrophy in patients with DM. Several studies have reported a reduction in the volume of the bilateral putamen in patients with both type 1 and type 2 DM (Antal et al. 2022; Habes et al. 2023; Jing et al. 2023). Structural changes in the putamen associated with DM may contribute to decreased spontaneous activity in the context of CKD. Despite findings by Wu et al. that the precuneus network is more active in patients with T2DM without cognitive impairment (2022), our results suggest that neuronal activity in the right precuneus may experience functional deterioration due to prolonged dual stressors in the context of CKD combined with DM. This difference may represent a critical transition from compensation to decompensation in disease progression.

To elucidate the interactions among distinct brain regions, FC offers a systematic method that allows researchers to evaluate connectivity patterns of neural activity (Zhang et al. 2021). We selected the bilateral putamen as seed points for FC analysis based on significant ALFF differences across groups and our prior research findings (Yu et al. 2024). All brain regions with statistically significant differences exhibited decreased FC, suggesting that CKD may disrupt neural communication. Consistent with the findings of Ding et al., we observed reduced FC between the right putamen and left SMA (Ding et al. 2018). The SMA is canonically involved in motor planning, coordination, and action sequencing. Beyond that, the SMA appears to participate in the brain's negative motor network, a circuit involved in inhibiting motor actions during execution (Pinson et al. 2022). Ding D et al. proposed that this reduced FC may constitute a neural mechanism underlying sensory dysfunction and involuntary leg movements in patients with CKD (Ding et al. 2018). Notably, decreased FC involving the putamen was observed. The putamen, a critical nucleus that makes up the basal ganglia, is primarily involved in the regulation of motor control and procedural learning (Ghandili and Munakomi 2025). Using a graph‐theoretical approach, Song L et al. found that the degree centrality of non‐dialysis patients with CKD stage 5 primarily decreased in the basal ganglia, which in the bilateral putamen was positively correlated with MoCA scores (Song et al. 2023). Another study using quantitative susceptibility mapping (QSM) and arterial spin labeling (ASL) demonstrated significant iron deposition and cerebral blood flow (CBF) alterations in the putamen of hemodialysis patients (Wang, Song, et al. 2023). All these findings provide evidence of functional alterations in the putamen. Taken together, our findings suggest that the putamen may be a critical target in CKD patients. Furthermore, our study revealed significantly reduced FC between the right putamen and right thalamus in the DKD group compared with the non‐DKD group. Emerging neuroimaging evidence implicates the thalamus as a vulnerable neuroanatomical structure, with observed alterations in both CKD and DM patients. Wang H et al. found reduced susceptibility values in the thalamus of patients with stage 5 CKD (Wang et al. 2023), while Jing et al. identified decreased thalamic volume in prediabetes, indicating early neural alterations prior to diabetes diagnosis (2023). We suggest that the thalamus undergoes dual pathological influences from CKD and DM, potentially serving as a target to distinguish DKD from non‐DKD patients.

In our study, we observed correlations between the FC using bilateral putamen as seed points and some laboratory indicators in both the non‐DKD and DKD groups. In the non‐DKD group, FC between the right putamen and left SMA exhibited a negative correlation with PTH, whereas in the DKD group, serum calcium showed a positive correlation with FC between the right putamen and right thalamus. Secondary hyperparathyroidism and hypocalcemia are prevalent among patients with CKD. Potential detrimental impacts of PTH on the central nervous system have been proposed (Duque et al. 2020). Our results suggest a potential impact of elevated PTH levels and hypocalcemia on spontaneous brain activity. Proteinuria and a state of micro‐inflammation are key clinical features of CKD and are strongly associated with disease complications (Liu et al. 2022; Sarnak and Astor 2011). Elevated urinary protein excretion often leads to hypoalbuminemia. Notably, in the non‐DKD group, FC between the right putamen and right thalamus exhibited a positive correlation with UPCR, while FC between the left putamen and right putamen showed a positive correlation with CRP. These findings suggested potential compensatory mechanisms in response to early neural adaptations. Conversely, this compensatory relationship was absent in the DKD group (ALB positively correlated with the FC between right putamen and right thalamus; ESR and CRP negatively correlated with the FC between right putamen and right caudate), possibly indicating a shift toward decompensation. Anemia (HGB) emerged as a key factor influencing FC in the DKD group. A previous study has shown that serum HGB levels, which exhibit a negative correlation with renal fibrosis, may serve as an accessible predictor for early DKD progression (Yamanouchi et al. 2022). We identified three distinct FC patterns that exhibited significant positive correlations with HGB levels in the DKD group: the FC between the right putamen and right thalamus, the right putamen and right caudate, and the left putamen and right putamen. However, no such associations were observed in the non‐DKD group. The more pronounced positive correlation between HGB levels and the FC involving the putamen in the DKD group suggests a heightened sensitivity of these neural circuits to variations in oxygen‐carrying capacity. These findings potentially indicate a greater vulnerability to hypoxia stress within the pathophysiological context of diabetic microangiopathy and uremia. Our findings suggest that patients with DKD may derive clinical benefits from anemia management in neurological function improvement. Although the cardiovascular and renal benefits of treating anemia in DKD are well‐established (Tsai and Tarng 2019), additional real‐world evidence is required to elucidate its impact on neurological function.

This study has several limitations. First, its cross‐sectional and observational design precludes causal inferences. Second, the small sample size may limit the generalizability of the findings. Third, the absence of cognitive assessments (e.g., MoCA scores) restricts the exploration of the relationship between cognitive function and brain function in CKD patients. Fourth, despite the FDR correction (*q* < 0.05), the extensive testing suggests that a small proportion of the identified effects may represent false discoveries. Future research should employ large‐scale, longitudinal studies to validate these findings.

## 5 Conclusions

In summary, this study employed ALFF and FC analyses to examine alterations in spontaneous brain activity among patients with non‐DKD, DKD, and HCs. The results demonstrated that both the non‐DKD and DKD groups displayed significant alterations in functional characteristics compared to the HC group, such as reduced ALFF values in the bilateral putamen and elevated ALFF values in the left middle occipital gyrus. The FC exhibited a statistically significant reduction in both the non‐DKD and DKD groups. Furthermore, the DKD group exhibited significantly lower ALFF values in the left putamen and right precuneus, as well as reduced FC between the right putamen and right thalamus, compared to the non‐DKD group. Correlation analysis revealed that several clinical laboratory indices, including UPCR, CRP, and HGB, exhibited significant correlations with the FC between the putamen and specific brain regions. Overall, these findings reveal the alterations of functional patterns in patients with CKD, with distinct patterns between non‐DKD and DKD subgroups.

## Author Contributions

**Xiwen Lei**: writing – original draft, writing – review and editing, visualization. **Yiyan Sun**: methodology, software, validation, formal analysis, data curation, visualization. **Juan Li**: conceptualization, supervision, project administration, funding acquisition. **Shuang He**: methodology, software. **Yangjie Yu**: methodology, software. **Xuyun Hua**: conceptualization, resources, supervision, project administration, funding acquisition. **Junjie Pan**: conceptualization, supervision, project administration, funding acquisition. **Rui Dong**: conceptualization, investigation, resources, data curation, writing – review and editing, supervision, project administration, funding acquisition. **Jianguang Xu**: conceptualization, supervision, project administration, funding acquisition.

## Funding

This research was funded by the Shanghai Municipal Health Commission Project (202140506).

## Ethics Statement

The study was conducted in accordance with the Declaration of Helsinki, and approved by the the Ethics Review Board of Shanghai Changhai Hospital, Naval Medical University (No. CHEC2022‐159).

## Consent

All enrolled participants completed the informed consent form.

## Conflicts of Interest

The authors declare no conflicts of interest.

## Data Availability

The datasets generated or analyzed during this study are available from the corresponding author on reasonable request.
